# Extracapsular extension is not a significant prognostic indicator in non-squamous cancers of the major salivary glands

**DOI:** 10.1186/s41199-018-0032-x

**Published:** 2018-07-03

**Authors:** Shayan Cheraghlou, Phoebe K. Yu, Michael D. Otremba, Saral Mehra, Wendell G. Yarbrough, Benjamin L. Judson

**Affiliations:** 1 Division of Otolaryngology, Department of Surgery, Yale School of Medicine, 800 Howard Ave., YPB 425, New Haven, CT 06519 USA; 2 Yale Cancer Center, New Haven, CT USA; 3 Department of Pathology, Yale School of Medicine, New Haven, CT USA

**Keywords:** Salivary gland cancer, ECE, Prognostic factor, Survival, NCDB

## Abstract

**Background:**

Extracapsular extension (ECE) is a well-established prognostic feature in squamous cell cancers of the head and neck. Although some extrapolate data from mucosal head and neck cancer to include ECE as a high-risk feature in salivary gland cancers, data is lacking about ECE’s prognostic value for these malignancies. We investigate whether ECE is a significant prognostic indicator in pathologic node-positive cancers of the major salivary glands.

**Methods:**

A retrospective study of adult salivary gland cancer cases diagnosed from 2004 to 2013 in the NCDB was conducted. Demographic, tumor, treatment, and survival variables were included in the study. Univariate Kaplan-Meier analyses, as well as multivariate Cox survival regressions were performed.

**Results:**

Positive ECE status was associated with significantly worse survival in salivary SCC (HR 1.687; *p* = 0.002) but not non-squamous salivary cancers (HR 1.000; *p* = 0.998) on multivariate analysis. While post-operative radiotherapy was not associated with improved survival for patients without high-risk adverse features (high grade or positive surgical margins), its use was associated with better survival for ECE-positive salivary SCC patients without one of these additional adverse features (HR 0.064; *p* = 0.010).

**Conclusions:**

Although ECE is a significant prognostic indicator in salivary SCC, its prognostic significance for non-squamous salivary cancers may be limited. Radiotherapy may improve survival in cases with at least one high-risk adverse feature: high grade; positive surgical margins; and for salivary SCC specifically, positive ECE status.

## Background

Cancers of the major salivary glands are rare malignancies estimated to have an incidence of 1.7/100,000 [1], composing about 5% of cancers of the head and neck [2]. Due to this rarity, its study has proven difficult, leading some to apply their knowledge of more common head and neck cancers to their approach to salivary malignancies. One of the clearest examples of this is the utilization of extracapsular extension (ECE) for these cancers. ECE is among the most important negative prognostic factors for cancers of the head and neck, with two randomized trials (EORTC-22931, RTOG-9501) clearly demonstrating its prognostic value [3]. However, these studies, like many of the others that demonstrate this association, included only patients with upper aerodigestive tract cancers [3–9]. The strength of these studies has led some to extrapolate the data to include ECE as a negative prognostic indicator for salivary gland cancers.

This extrapolation is not espoused by national guidelines [10] and there are limited data supporting the practice. It is particularly problematic due to the significant differences between salivary gland cancers and squamous cell head and neck malignancies. Salivary gland cancers have a large degree of histological heterogeneity, with over 20 identified malignant variants, the majority of which are non-squamous salivary cancers [11]. While salivary squamous cell carcinomas (SCC) can occur in the major salivary glands, many argue that the majority are metastases from cutaneous squamous cell carcinomas rather than primary salivary malignancies [12, 13]. This underlying biological difference between salivary gland cancers and upper aerodigestive tract malignancies may lead to different behavior and ECE status may not be as indicative of aggressiveness for salivary gland malignancies as it is for upper aerodigestive tract cancers. Small single-institution reviews have noted a trend towards poorer survival for ECE-positive (ECE+) salivary gland cancers of particular histologic subtypes or stages [14–16], but there has yet to be a large-scale study indicating ECE’s prognostic value for salivary gland cancers.

Additionally, ECE status may have implications for treatment decision-making. Analysis of EORTC-22931 and RTOG-9501 revealed that patients with certain adverse features, including positive ECE status had improved survival with the addition of chemotherapy to adjuvant radiotherapy [3, 17]. The National Comprehensive Cancer guidelines suggest adjuvant radiotherapy after surgery for salivary gland cancers in the presence of adverse features, but ECE status is currently not included in this decision tree [10]. While the presence of any node metastases is considered an indication for radiotherapy, it is not utilized uniformly. Additionally, the benefit of adding chemotherapy to adjuvant radiotherapy for salivary malignancies is not established beyond palliative use [10]. It is unclear how ECE status affects outcomes following treatment with or without adjuvant therapy.

We aimed to determine the prognostic significance of ECE status in patients with node-positive cancers of the major salivary glands. We had a secondary aim of determining if this significance varied by histology and whether ECE status impacted the survival benefit associated with adjuvant therapies. To accomplish this, we examined a cohort of 1472 patients from the Commission on Cancer’s National Cancer Data Base (NCDB) who were diagnosed between 2004 and 2013 inclusive.

## Methods

### Data source

Data originated from the National Cancer Database (NCDB) from 2004 to 2016. The NCDB is a nationwide clinical surveillance resource data set that includes approximately 70% of all newly diagnosed malignancies in the United States from over 1500 cancer programs as previously described [18]. This data source has been commonly utilized in the head and neck cancer literature [19–24]. This study was determined to be exempt from institutional review by the Yale Human Investigation Committee.

### Study population

**Fig. 1:**
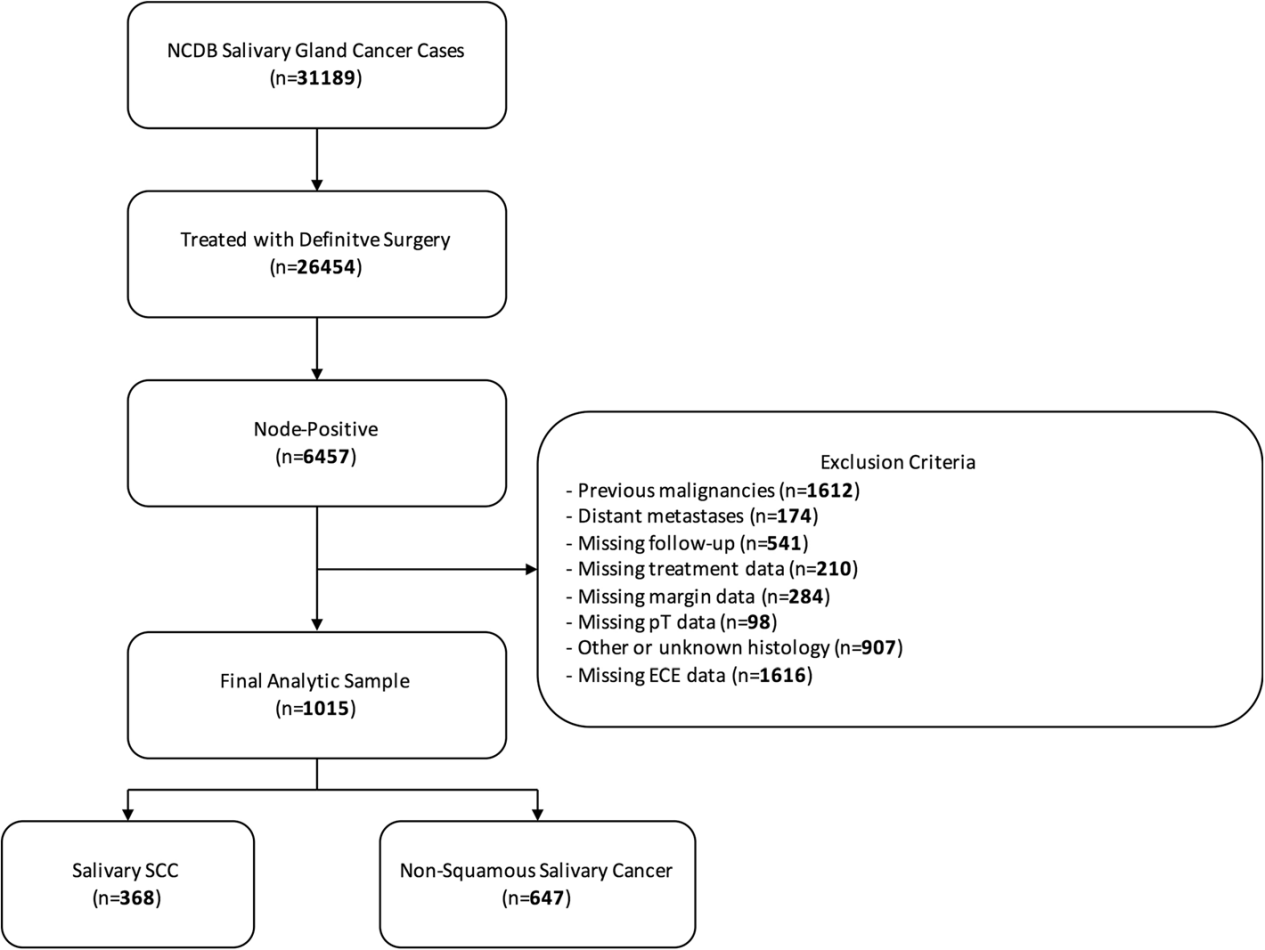
CONSORT diagram of patient selection

Our selection criteria are presented in Fig. 1. We identified cases with a primary site in the parotid, submandibular, or sublingual glands by the International Classification of Disease for Oncology, 3rd Edition (ICD-O-3) topography codes C07.9 – parotid gland (*n* = 849), C08.0 – submandibular gland (*n* = 131), C08.1 – sublingual gland (*n* = 8), C08.8 – overlapping lesion of major salivary glands (*n* = 2), C08.9 – major salivary gland, NOS (*n* = 25). We classified patients by ICD-O-3 histology codes 8430 (mucoepidermoid), 8550 (acinic cell), 8200 (adenoid cystic), 8140 (adenocarcinoma), and 8940/8941 (malignant mixed) as non-squamous salivary cancers and 8050–8083 (squamous cell) as salivary SCC. We included pathologic node-positive adult patients who were treated with definitive surgery. We excluded patients if they had other primary malignancies; had distant metastases; had a histologic subtype other than salivary SCC or the included non-squamous salivary cancers subtypes; or had missing follow-up, pathologic T stage, treatment, ECE, or margin data.

### Statistical analysis

Patient, tumor, and treatment variables were included in the analysis. Comorbidity was measured by the Charlson/Deyo score, with 0 corresponding to no comorbidity; 1 to cardiovascular disease, dementia, chronic pulmonary disease, rheumatologic disease, peptic ulcer disease, mild liver disease, or diabetes; and 2 or greater to diabetes with chronic complications, hemiplegia or paraplegia, renal disease, moderate or severe liver disease, or AIDS. Pathologic staging was based on American Joint Committee on Cancer (AJCC) 7th edition criteria. Surgical margins were defined as positive if there was any evidence of residual disease. Patients were considered to have received surgery if they received any definitive surgical procedure at the primary site: local tumor excision, partial parotidectomy or removal of major salivary gland, total parotidectomy or removal of major salivary gland, radical parotidectomy or removal of major salivary gland, or parotidectomy, not otherwise specified (NOS). Patients were defined as having received chemotherapy if they received any chemotherapy, regardless of the type or number of agents. Patients were defined as having received radiotherapy if they received external beam radiation with or without other radiotherapy. Extracapsular extension status was defined as positive if there was any pathologic evidence of extracapsular extension, either microscopic or macroscopic.

Chi-square analyses were performed to determine differences in study variables by patient ECE status. Multivariate Cox survival regressions were completed in order to determine the prognostic significance of ECE status by tumor histology. Step-wise removal of factors found to be insignificant was then performed in order to account for multicollinearity. This model was used to produce multivariate adjusted survival curves by tumor histology and ECE status. Multivariate Cox regressions and univariate Kaplan-Meier analyses were conducted in order to determine differences in survival by the presence of ECE and other high-risk adverse features (positive margins and high grade). Finally, multivariate Cox regressions were performed to determine the survival advantage associated with adjuvant therapies stratified by ECE status and tumor histology. Statistical significance was determined at the *p* < 0.05 level. All data analysis was performed using STATA version 13 (StataCorp LP, College Station, TX).

## Results

The characteristics of the study population are described in Table 1. The majority of patients were male (68.1%), white (84.0%), and had no comorbidities (79.7%). Most had tumors with a T stage of 3 or 4 (62.6%), an N stage of 2 (62.4%), and a high grade (58.1%). The non-squamous salivary cancer group contained 174 mucoepidermoid carcinomas, 79 acinic cell carcinomas, 111 adenoid cystic carcinomas, 200 adenocarcinomas, and 83 malignant mixed carcinomas. Adjuvant administration of radiotherapy (77.1%) was more common than chemotherapy (33.5%). Patients with extracapsular extension were more commonly male, and tended to have higher grade and higher T/N stage disease. They were also more likely to have positive surgical margins and be treated with radiotherapy and/or chemotherapy in addition to definitive surgery.

**Table 1:** Characteristics of the study population

Variable, *p*-value of chi-square^a^	No. (%)		
	Total (*n* = 1015)	ECE Negative (*n* = 611)	ECE Positive (*n* = 404)
Age, *p* = 0.609			
≤54	226 (22.3)	140 (22.9)	86 (21.3)
55–64	264 (26.0)	151 (24.7)	113 (28.0)
65–74	234 (23.0)	139 (22.8)	95 (23.5)
≥75	291 (28.7)	181 (29.6)	110 (27.2)
Sex, *p* = 0.009			
Female	324 (31.9)	214 (35.0)	110 (27.2)
Male	691 (68.1)	397 (65.0)	294 (72.8)
Race, *p* = 0.273			
White	853 (84.0)	508 (83.1)	345 (85.4)
Black	80 (7.9)	52 (8.5)	28 (6.9)
Hispanic	46 (4.5)	25 (4.1)	21 (5.2)
Asian/Pacific Islander	27 (2.7)	21 (3.4)	6 (1.5)
Other/Unknown	9 (0.9)	5 (0.8)	4 (1.0)
Charlson/Deyo Score, *p* = 0.815			
0	809 (79.7)	491 (80.4)	318 (78.7)
1	156 (15.4)	91 (14.9)	65 (16.1)
≥2	50 (4.9)	29 (4.8)	21 (5.2)
Pathologic T Stage, *p* < 0.001			
1	134 (13.2)	101 (16.5)	33 (8.2)
2	246 (24.2)	167 (27.3)	79 (19.6)
3	347 (34.2)	192 (31.4)	155 (38.4)
4	288 (28.4)	151 (24.7)	137 (33.9)
Pathologic N Stage, p < 0.001			
1	373 (36.8)	296 (48.4)	77 (19.1)
2	633 (62.4)	312 (51.1)	321 (79.5)
3	9 (0.9)	3 (0.5)	6 (1.5)
Grade, *p* = 0.001			
Low	64 (6.3)	48 (7.9)	16 (4.0)
Intermediate	191 (18.8)	130 (21.3)	61 (15.1)
High	590 (58.1)	328 (53.7)	262 (64.8)
Unknown	170 (16.8)	105 (17.2)	65 (16.1)
Surgical Margins, *p* < 0.001			
Negative	553 (54.5)	363 (59.4)	190 (47.0)
Positive	462 (45.5)	248 (40.6)	214 (53.0)
Histology, *p* = 0.736			
Squamous Cell	368 (36.3)	219 (35.8)	149 (36.9)
Non-Squamous Cell	647 (63.7)	392 (64.2)	255 (63.1)
Radiotherapy, *p* = 0.003			
Not Administered	232 (22.9)	159 (26.0)	73 (18.1)
Administered	783 (77.1)	452 (74.0)	331 (81.9)
Chemotherapy, *p* < 0.001			
Not Administered	675 (66.5)	451 (73.8)	224 (55.4)
Administered	340 (33.5)	160 (26.2)	180 (44.6)

**Fig. 2:**
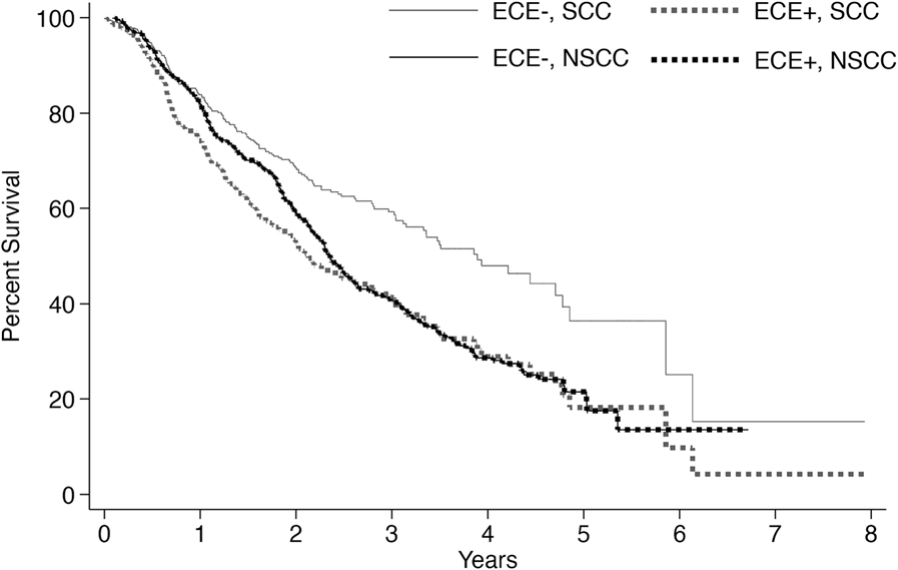
Multivariate adjusted survival functions stratified by histology and ECE status. SCC – Squamous Cell Carcinoma, NSCC – Non-Squamous Cell Carcinoma

The prognostic significance of ECE varied based on tumor histology (Table 2). Among patients with salivary SCC, positive ECE status was significantly associated with worse survival (Hazard Ratio [HR], 1.449; *p* = 0.048). However, among patients with non-squamous salivary cancer, ECE status was not associated with a difference in survival (HR 1.000; *p* = 0.998). These results were unchanged after stepwise removal, although the association of ECE with poorer survival among patients with salivary SCC proved more significant (HR 1.687; *p* = 0.002). Multivariate adjusted survival curves stratified by histology and ECE are presented in Fig. 2. Among patients with salivary SCC, 3-year survival rates for ECE- and ECE+ cases were 63.8% (SE: 3.6) and 44.8% (SE: 4.9) respectively, while 5-year survival rates were 41.1% (SE: 6.3) and 23.9% (SE: 10.4) respectively.

**Table 2:** Multivariate analysis of factors associated with survival

Variables	Squamous Cell		Non-Squamous Cell	
	Hazard Ratio	*p*-value	Hazard Ratio	*p*-value
Age				
≤54	1 [Reference]	–	1 [Reference]	–
55–64	1.105	0.778	1.313	0.154
65–74	1.353	0.377	1.467	0.054
≥75	2.342	0.006	2.165	< 0.001
Sex				
Female	1 [Reference]	–	1 [Reference]	–
Male	0.987	0.949	1.051	0.723
Race				
White	1 [Reference]	–	1 [Reference]	–
Black	0.871	0.768	1.162	0.443
Hispanic	1.908	0.113	0.462	0.091
Asian/Pacific Islander	2.007	0.148	0.386	0.184
Other/Unknown	0.000	1.000	1.628	0.351
Charlson/Deyo Score				
0	1 [Reference]	–	1 [Reference]	–
1	1.406	0.093	1.430	0.046
≥2	1.569	0.153	2.395	0.001
Pathologic T Stage				
1	1 [Reference]	–	1 [Reference]	–
2	1.798	0.070	1.583	0.127
3	1.791	0.059	2.998	< 0.001
4	2.781	0.001	3.268	< 0.001
Pathologic N Stage				
1	1 [Reference]	–	1 [Reference]	–
2	1.403	0.077	1.261	0.144
3	0.876	0.897	0.565	0.443
Grade				
Low	1 [Reference]	–	1 [Reference]	–
Intermediate	1.090	0.824	1.554	0.320
High	0.719	0.389	2.786	0.009
Unknown	0.617	0.306	2.471	0.028
Surgical Margins				
Negative	1 [Reference]	–	1 [Reference]	–
Positive	1.350	0.086	1.093	0.505
Radiotherapy				
Not Administered	1 [Reference]	–	1 [Reference]	–
Administered	0.559	0.005	0.622	0.003
Chemotherapy				
Not Administered	1 [Reference]	–	1 [Reference]	–
Administered	0.779	0.236	1.309	0.064
ECE Status				
Negative	1 [Reference]	–	1 [Reference]	–
Positive	1.449	0.042	1.000	0.998

**Fig. 3:**
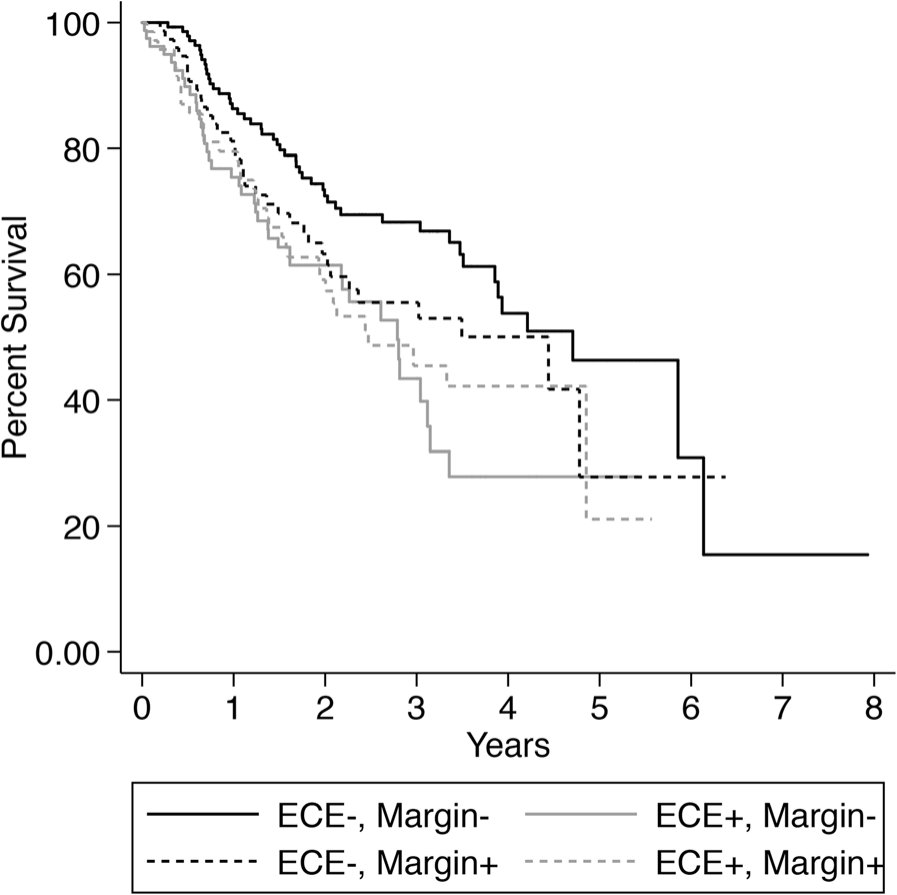
Kaplan-Meier survival stratified by ECE status and margin status for patients with salivary SCC

The prognostic significance and relationship of extracapsular extension with two other established adverse factors, high grade and positive margins, were also explored. Among patients with salivary SCC, positive ECE status was associated with diminished survival in cases with negative margins (HR 1.961; *p* = 0.004) but not in cases with positive margins (HR 1.033; *p* = 0.915), while positive margins were associated with diminished survival in ECE- cases (HR 1.813; *p* = 0.015) but not in ECE+ cases (HR 1.194; *p* = 0.509). Regardless of the status of the other adverse feature, neither ECE status nor margin-status were associated with survival for patients with non-squamous salivary cancer. Among patients with salivary SCC, presence of either ECE or high-grade was associated with significantly worse survival. However, presence of both features was not associated with poorer survival than either the presence of ECE or high-grade exclusively. Among patients with non-squamous salivary cancer, high-grade (HR 2.786; *p* = 0.009), but not positive ECE status (HR 1.000; *p* = 0.998), was associated with diminished survival (Table 2). Among patients with SCC, 3-year survival was 68.3% (SE: 4.3) for cases that were both ECE and margin negative and 45.4% (SE: 6.9) for cases that were both ECE and margin positive. Survival by joint margin/ECE status among patients with salivary SCC is presented in Fig. 3.

The benefit of additional therapy varied by histology and the presence of adverse features, including ECE. Radiotherapy was associated with improved survival among both salivary SCC (HR 0.556; *p* = 0.009) and non-squamous salivary cancer (HR 0.652; *p* = 0.012) cases with at least one additional high-risk adverse feature, either high grade or positive surgical margins. In the absence of these high-risk adverse features, radiotherapy was not associated with improved survival for either salivary SCC or non-squamous salivary cancer. However, among ECE+ salivary SCC cases, radiotherapy was associated with improved survival even in the absence of high grade or positive surgical margins (HR 0.064; *p* = 0.010). Treatment with chemotherapy was not associated with improved survival, regardless of the presence of ECE for both salivary SCC and non-squamous salivary cancer.

## Discussion

The prognostic value of ECE has been well established in upper aerodigestive tract cancers [3–9]. However, evidence has been limited for ECE’s use in cancers of the salivary glands, which vary both histologically and behaviorally from the aforementioned mucosal cancers [14–16]. Studies of non-head and neck malignancies, including those of other glandular tissues, demonstrate that the significance of ECE as a prognostic factor varies by site [25–33]. In the present study, we found that ECE was significantly prognostic of survival in salivary SCC but not non-squamous salivary cancers.

We found that ECE may be utilized as an independent prognostic factor in salivary SCC. Our results suggest that the presence of ECE or other established high-risk adverse features (high grade or positive surgical margins) [34–40] is predictive of poorer survival. In fact, positive-ECE status was associated with worse survival than positive surgical margins. This set of adverse post-operative features is similar to that which is utilized for mucosal cancers of the head and neck [3, 41, 42]. However, despite some suggestion that ECE trends towards significance as a prognostic indicator for salivary gland malignancies of certain histologies or stages in the literature [14–16], after controlling for other demographic and clinical factors, we found that ECE showed no prognostic significance for non-squamous salivary cancers.

Additionally, we found that the survival benefit of post-operative radiotherapy varied by tumor histology and the presence of ECE and other high-risk adverse features. As has been previously demonstrated [34–36, 43], we found that the addition of adjuvant radiotherapy to the post-operative care of patients with salivary cancers was associated with a marked survival benefit. This effect persisted regardless of tumor histology when an established high-risk feature, high grade or positive surgical margins, was present. In the absence of these features, adjuvant radiotherapy was no longer associated with improved survival for both salivary SCC and non-squamous salivary cancer cases. For ECE+ salivary SCC cases however, radiotherapy was associated with a survival benefit even in the absence of high grade or positive surgical margins. Our data suggest that, as is outlined in UK guidelines for salivary gland cancer management [44], post-operative radiotherapy may not be required for patients who do not have the previously outlined high-risk features.

Addition of chemotherapy to adjuvant radiotherapy for salivary cancers has also been recently debated. Current National Cancer Comprehensive Network guidelines suggest that while added chemotherapy may be considered in some high-risk features, data is too sparse to definitively recommend its use in these cases. In fact, recent work suggests that chemotherapy may not provide a significant survival benefit in the adjuvant treatment of salivary malignancies [45–48]. We found that both in the presence and absence of ECE, high-grade, and positive surgical margins, chemotherapy was not associated with improved survival regardless of tumor histology. We anticipate that the RTOG-1008 trial will bring further clarity to this issue. For salivary SCC in particular, the RTOG-0501 trial, studying the efficacy of adjuvant chemotherapy for high-risk cutaneous SCC of the head and neck may provide some direction for future treatment, given that the majority of these tumors are metastases from a primary cutaneous malignancy.

Our study had several limitations. Firstly, we were unable to control our analysis for a number of features (ie. perineural invasion, nodal location, molecular markers) that may have also contributed to survival, either due to extensive missing data or their absence from the data source. Furthermore, the association of ECE with several other adverse features presents a possible selection bias. Nonetheless, ECE remained a significant prognostic factor when controlling for these other features. Additionally, although the majority of salivary SCC’s likely originate from a primary cutaneous malignancy, the lack of data collection on non-melanoma skin cancers in national registry data limits our ability to differentiate between these more common types of salivary SCC and the more rare primary salivary SCC. Finally, the lack of information about the chemotherapy agents utilized limits our ability to draw conclusions about the efficacy of specific chemotherapy regimens.

## Conclusion

This is, to our knowledge, the first study to explore the prognostic significance of ECE by histologic subtype in cancers of the major salivary glands. Our data suggest that ECE may not be an accurate prognostic indicator in non-squamous salivary cancer, although further work is required in order to confirm this suggestion. Our results also suggest that the addition of ECE to established high-risk adverse features for salivary SCC might provide more accurate prognoses during patient counseling and treatment planning. Additionally, our study indicates that the presence of ECE may suggest a more significant benefit to post-operative radiotherapy in salivary SCC cases.

## Abbreviations

ECE: Extracapsular extension
SCC: Squamous cell carcinoma
